# Heterochronic parabiosis: a valuable tool to investigate cellular senescence and other hallmarks of aging

**DOI:** 10.18632/aging.204015

**Published:** 2022-04-13

**Authors:** Matthew J. Yousefzadeh, Paul D. Robbins, Derek M. Huffman

**Affiliations:** 1 Institute on the Biology of Aging and Metabolism, University of Minnesota, Minneapolis, MN 55455, USA; 2 Department of Biochemistry, Molecular Biology, and Biophysics, University of Minnesota, Minneapolis, MN 55455, USA; 3 Department of Molecular Pharmacology and Medicine, Albert Einstein College of Medicine, Bronx, NY 10461, USA; 4 Institute for Aging Research, Albert Einstein College of Medicine, Bronx, NY 10461, USA

**Keywords:** aging, cellular senescence, heterochronic parabiosis, geroscience

## Abstract

Parabiosis is a well-established method to facilitate a shared blood supply between two conjoined animals. In particular, the pairing of mice of dissimilar ages, termed heterochronic parabiosis, has been used extensively for differentiating cell autonomous and non-autonomous mechanisms of aging. Analysis of heterochronic parabionts also has helped to identify individual circulating factors that may act as either pro- or anti-geronics. Heterochronic parabiosis also has proven to be a valuable experimental system to evaluate the effects of specific hallmarks of aging on the process of aging. For example, heterochronic parabiosis was used recently to examine whether cellular senescence was driven via cell autonomous and/or non-autonomous mechanisms. As anticipated, markers of cellular senescence were elevated in old isochronically-paired mice relative to young controls. However, compared to old isochronically paired mice, the senescent cell burden was reduced in multiple tissues of old parabionts joined with young mice. This suggests that the rejuvenation of cells and tissues in old mice by exposure to young blood could be mediated, in part, through suppression or immune clearance of senescent cells. Conversely, young heterochronic parabionts showed increased markers of cellular senescence, demonstrating that exposure to an old circulation is able to drive senescence through a cell non-autonomous mechanism(s), likely contributing to accelerated aging in the young mice. Thus, heterochronic parabiosis is still an important methodology that should continue to be leveraged for evaluating other hallmarks of aging and their mechanisms.

## INTRODUCTION

Rodent models of aging serve as valuable tools to investigate mechanisms of aging and rejuvenation. However, differentiating cell autonomous from cell-nonautonomous aspects of aging in rodent models has proven difficult. Parabiosis has been an invaluable method to study the cell-nonautonomous effects of circulating factors shared between two surgically paired animals. This technique was pioneered by Paul Bert in the 1860s and remains in use to this day [1]. Of particular interest to the field of aging is the use of heterochronic parabiosis (HP) where a young and old mouse are paired together to form a shared blood supply. Over the past two decades, HP has demonstrated that exposure to the circulating milieu of young mice can rejuvenate many biological processes in old mice, including bone regrowth, hepatogenesis, myogenesis, and neurogenesis [2–10]. Interestingly, these features have been mechanistically linked in some reports to specific blood-borne proteins, termed anti-geronic factors as well as putative pathways involved. Conversely, HP also has been used to identify pro-geronic factors found in old mice that are able capable of driving aging in young heterochronic parabionts [3, 6, 8, 10, 11]. These seminal findings from the HP field have led to creation of companies to either sell “young blood” or test the efficacy of young plasma or enriched plasma fraction transfusions on age-related chronic diseases. Thus, HP provides a useful approach to assess the effects of the young and old environment, respectively, on the hallmarks of aging.

One hallmark of aging is cellular senescence [12, 13], a cell fate elicited in response to external and internal cellular stress signals, established through transcription factor cascades that can include p16^INK4a^/retinoblastoma protein and/or p53/p21^CIP1^, which causes extensive changes in gene expression, histone modifications, organelle function, elevated protein production, and profound morphologic and metabolic shifts. A significant fraction of senescent cells release inflammatory factors, chemokines, growth factors, proteases, bioactive lipids, prostenoids, extracellular vesicles, and pro-coagulant factors, termed the senescence-associated secretory phenotype or SASP that has been hypothesized to drive peripheral or secondary senescence *in vivo*. In a study from our group, HP was used to investigate the cell non-autonomous effects on the senescent cell burden in mice. This study measured markers of cellular senescence and SASP factors in multiple tissues from 6- or 20-month-old mice that were isochronically or heterochronically paired for two months [14]. As expected, expression of classical senescence markers *p16^Ink4a^* and *p21^Cip1^* were significantly higher in some tissues (forebrain, kidney, liver, lung, and pancreas) of older isochronically paired (OO) mice, compared to young isochronic parabionts (YY). *p16^Ink4a^* and *p21^Cip1^* expression was reduced in the tissues of old heterochronic parabionts (OY) compared to the old isochronically paired (OO) mice. Surprisingly, there was a concomitant increase in the expression of *p16^Ink4a^* and *p21^Cip1^* in tissues of young heterochronic parabionts (YO) in comparison to their isochronic controls (YY). Similar patterns of expression were observed for select SASP factors (*Il1ß*, *Il6*, *Mcp1*, and *Tnfa*) in the same tissues. The protein levels of MCP-1, a chemokine responsible for monocyte recruitment and a surrogate biomarker of biological aging [15, 16], were quantified in the kidneys and livers of HP mice. MCP-1 protein was reduced in old heterochronic parabionts (OY) and increased in young heterochronic parabionts (YO) relative to their respective controls (YY/OO) [14]. These changes in senescence were corroborated by composite lesion scores in some tissues based upon assessment of age-related histopathologic legions using the Geropathology Grading Platform [14, 17–19]. These findings clearly illustrate that cellular senescence could be influenced by the systemic environment and that the rejuvenating effects observed with HP are possibly mediated through diminution of senescent cell burden. It is important to note, however, that circulating functional T or NK cells from the young parabiont could also be contributing to reducing the senescent cell burden by clearing the senescent cells in the old counterpart.

Our findings were consistent with the recently proposed saturating removal (SR) model in which the accumulation of senescent cells production rises linearly with age with senescent cells inhibiting their own removal [20]. In the future, the application of emerging single cell and spatial genomic methods to better detect and characterize senescent cells will provide a better mechanistic understanding of this cell non- autonomous regulation of the senescent cell burden with age. Initial multiomic analysis of cells and tissues from HP mice revealed beneficial changes in transcriptome and epigenetic clock of old heterochronic parabionts, suggesting that exposure to youthful factors is sufficient to confer some reversal of the biological clock [21]. Furthermore, these positive effects persisted in the old HP mice for up to two months post-separation. Future experiments could include the use of genetic models for depletion of p16^INK4a+^ and p21^CIP1+^ senescent cells in HP parabionts to investigate the role of senescent cells in driving the increase in senescent cells and loss of tissue homeostasis in young mice. In summary, this established method of studying the role of circulating factors in cell and tissue rejuvenation by HP is still relevant and can be used to address many key questions in aging.
